# Design and optimization of a high efficiency CdTe–FeSi_2_ based double-junction two-terminal tandem solar cell

**DOI:** 10.1016/j.heliyon.2024.e27994

**Published:** 2024-03-12

**Authors:** Mehedi Hasan Tonmoy, Sheikh Noman Shiddique, Ahnaf Tahmid Abir, Jaker Hossain

**Affiliations:** Solar Energy Laboratory, Department of Electrical and Electronic Engineering, University of Rajshahi, Rajshahi, 6205, Bangladesh

**Keywords:** CdTe-FeSi_2_, Double-junction, Tandem PV cell, Current alignment, SCAPS-1D

## Abstract

This article theoretically demonstrates an enormously efficient CdTe–FeSi_2_ based dual-junction tandem solar cell accompanied by slender semiconductor layers. The peak efficiency of the device has been ensured through the optimization of its various attributes of window, CdTe (bandgap 1.5 eV) top absorber, FeSi_2_ (bandgap 0.87 eV) bottom absorber and back surface layers. Additionally, the impacts of thickness, doping and the level of defects in different window, base and rear surface layers have been examined to observe how different layers affect the solar cell's performance. The optimized n-CdS/p-CdTe/p^+^-MoS_2_--*n*-CdS/p-FeSi_2_/p^+^-Cu_2_SnS_3_ dual-junction tandem solar device displays an efficiency of 43.9% with a voltage at no load, V_OC_ of 1.928 V, current density under a closed circuit, J_SC_ of 25.34 mA/cm^2^, and fill factor of 89.88%, respectively. These results disclose the high potential of the suggested solar cell based on CdTe and FeSi_2_ compounds.

## Highlights

• Numerical analysis 2J 2-terminal CdTe–FeSi2 tandem solar cell has been performed.
• Defects in the absorbers and interfaces have been optimized to maximize the cell output.
• Optimized CdTe–FeSi_2_ tandem cell provides a V_OC_ = 1.928 V and an efficiency = 43.9%.
• These results are hopeful in the development highly efficient CdTe tandem solar cells.

## 1 Introduction

The technology of Tandem photovoltaic (PV) cell renders a hopeful path to significantly ameliorate the photovoltaic (PV) device efficiency. It involves combining two solar cell technologies: one with a larger bandgap (around 1.5–1.8 eV) on top of another with a smaller bandgap (∼0.9–1.2 eV). The top cell transforms photons with elevated energy efficiently while minimizing thermalization losses and transmitting the solar spectrum in the close-infrared region light to the lower cell. This approach has demonstrated success, achieving up to 31.1% power conversion efficiency in single crystals of III-V semiconductors solar cells under standard 1-sun illumination [1]. The critical question, however, is whether tandem solar cells can reach a levelized cost of electricity (LCOE) competitive enough for one-sun applications, given the higher fabrication costs associated with tandems. Furthermore, tandems of thin-film solar cells, where sub-cells share production methods and cost structures, provide economic benefits [2]. Efficient tandem solar cell design requires careful consideration of the electrical and visual attributes of each sub-cell to minimize losses from electrical mismatches [3]. Power matching, where each sub-cell operates at its peak power output with the same current, is challenging to achieve experimentally. Typically, researchers use current matching ensuring the sub-cells have matching short-circuit currents, often assessed through spectral response or quantum efficiency analysis [3]. It is important to note that the conditions for matching current and power may vary significantly, especially with sub-cells having markedly different fill factors [4]. Single-junction solar cells face limitations as they can solely capture photons with energy gaps that are equal to or exceed the bandgap of the materials they are made of. Tandem solar cells offer a solution by incorporating multiple sub-cells with various absorber materials, broadening their light absorption range [5]. The potential power conversion efficiency (PCE) of solar cells with multiple junctions in tandem surges as the number of junctions spreads. For instance, a double-junction cell achieves approximately 45% efficiency, while a triple-junction cell reaches around 51% efficiency [6]. However, the theoretical maximum efficiency of a tandem solar cell with an unlimited number of sub-cells reaches 68.2% [7]. These tandem cells can be created using different materials, effectively dividing the power spectrum among different absorbers, typically featuring a top sub-cell optimized for low-wavelength photon absorption and a bottom sub-cell for high-wavelength photons [8]. In multi-junction devices, there are two primary orientations: two-edges and four-edges devices. Devices having four edges depicts more complexity, requiring intricate electrical elements for incorporation into modules and systems. Two-terminal devices, while simpler, must ensure matching the current between the upper and lower cells while maintaining a high fill factor (FF) [9]. This study primarily aimed at double-terminal dual-junction devices with monolithically integrated layers. These devices stack two photovoltaic cells with absorber layers of semiconductors with varying energy bandgaps, positioning the cell with the material with the broadest bandgap positioned at the front side where irradiance occurs, minimizing thermalization losses. For the global mutation from single-junction to the technology of multi-junction solar cells, certain criteria need to be satisfied, including cost competitiveness, abundant and cost-effective raw materials, and stability and longevity of each junction and the entire device [10,11]. In the realm of multi-junction configurations, the monolithic integration of a two-terminal (MI-2T) tandem device stands out as the most feasible choice for economical, large-scale use. However, achieving MI-2T tandem devices in practice remains challenging due to the need for compatible processing steps without compromising preceding interfaces and layers [12].

In this work, Dual-junction two-terminal tandem cells based on Cadmium telluride (CdTe) and Iron di-Silicide (FeSi_2_) semiconductor have been designed and extensively investigated theoretically. To achieve optimal production from such a dual-junction tandem cell, the formula: E_g top_ = 0.5 × E_g bot_ +1.15 eV is essential to ensure, wherein E_g top_ represents the optical bandgap of the upper absorbing material, whereas E_g bot_ indicates the optical bandgap of the lower absorbing material, to select proper energy gaps [13]. Consequently, materials such as CdTe with an energy gap of 1.5 eV and FeSi_2_ with an energy gap of 0.87 eV have been pondered for the upper and lower absorbing materials, respectively.

CdTe thin-film photovoltaic technology made its debut in the early 1970s and today, it stands as the sole thin-film production ranked among the top ten globally [14]. This is attributed to the exceptional durability and strong chemical stability of CdTe. Moreover, these characteristics enable CdTe could be applied using a broad range of array of available methods, makes it well-suited for large-scale manufacturing [15]. CdTe belonging to II–VI heterojunction compound solar cells represent promising options for achieving efficient conversion of solar energy at a cheap rate [16]. Lately, CdTe has become a prominent absorber material in the field of thin-film solar cell applications, demonstrating impressive photoconversion efficiency exceeding 21.0% and a voltage in open-circuit exceeding 1 V, as evidenced by recent studies. Currently, solar cells utilizing CdTe as their base material hold a notable share of approximately 5.1% in the global photovoltaic market, particularly in the realm of Photovoltaic cells with thin layers, positioning themselves as the second most economical materials after silicon [17]. Furthermore, there are predictions that the effectiveness of solar cells focused on CdTe has the potential to be pushed even higher, potentially reaching 28.04%, through the incorporation of a NiO hole transport layer [18].

The elements iron (Fe) and silicon (Si) used to form FeSi_2_ are two of the most common elements have discovered in the Earth's outer shell [19]. As a result, FeSi_2_ proves to be a cost-effective material, making it a viable choice for use as an active absorber material in the manufacturing of solar photovoltaic (PV) panels. Also, FeSi_2_ is resistant to chemical changes at plentiful temperatures up to 937 °C, non-toxic and exhibits exceptional resistance to oxidation and moisture [20]. The carrier diffusion length of this is quite long about 38 μm [21]. The material's specific gravity of 4.93, places it in the margin of Si (2.33) and GaAs (5.33) in the density spectrum [22]. Additionally, FeSi_2_ provides strong resistance to cosmic and radioactive exposure [22]. FeSi_2_ films can be cultivated on a solid surface at standard room temperature (RT) [23]. FeSi_2_ can capture photons spanning from the visible spectrum to near-infrared (NIR) light to the maximum wavelength of 1675 nm [24]. These characteristics render it a fitting choice for third-generation (3G) solar photovoltaic (PV) technology, particularly in space applications [22]. FeSi_2_ can be used to manufacture solar cells, photo sensors, and generators that utilize the thermoelectric effect compatible for integration with large-scale Si (silicon) circuits [22].

In order to improve light absorption, it is crucial to reduce the undesired losses at the junction resulting from Fresnel surface reflection [25]. Cadmium sulfide (CdS) is a nanostructured material in high demand, extensively explored by researchers across the globe. Its wide energy bandgap, adjustable properties, abundant carrier concentration, remarkable light transmission, and stability under continuous light make it a subject of significant interest [26,27]. It is a recognized semiconductor of the n-type variety that can be manufactured in various forms such as films, powders, pellets, nanorods and nanowires [28]. So, a CdS layer with a thickness of fewer than 100 nm in thickness is desired for efficient CdTe solar cell fabrication. Consequently, when the CdS layer is thinned to just a few tens of nanometers, it is almost certain that a pinhole will form [29,30]. The process of depositing the CdS window layer involves the use of chemical bath deposition techniques [28]. The CdS semiconductor possesses a bandgap of 2.4 eV, along with energy levels of 4.30 eV for E_C_ and 6.7 eV for E_V_. Consequently, CdTe readily establishes a desirable pn junction with CdS [18].

The BSF layer diminishes light absorption non-uniformity and scatters photons identically throughout the absorber layer. The BSF layer can be used instead of the standard anti-reflecting coating (ARC) to enhance the acquiring of photons within photovoltaic cells. The other benefit of the BSF has been its ability to reduce the loss of recombination at the surface [31,32]. Molybdenum disulfide (MoS_2_) has been selected as the (BSF) of the top cell due to its ability to form heterojunctions with CdTe. MoS_2_ is typically grown using chemical vapor deposition. The material has an energy bandgap of 1.62 eV and a diffusion length of 1 μm. Furthermore, MoS_2_ has a coefficient for absorption of 10^4^ cm^−1^ making it an excellent sunlight harvester. This material is also cost-effective to produce [33,34]. During a prior study, MoS_2_ was utilized as the layer of hole transfer in conjunction using solar cells based on SnS resulting in a calculated efficiency of approximately 41% [34].

Copper Tin Sulfide, Cu_2_SnS_3_ (CTS) has been utilized as a BSF layer for the bottom cell. CTS consists of the elements that are non-toxic, found abundantly on Earth, and have documented bandgap values ranging from 0.93 to 1.35 eV [[35], [36], [37]] and the coefficient absorption is greater than 10^4^ cm^−1^ [37], making it as a potential choice for solar cells. Various methods for deposition, processes like spray pyrolysis, co-evaporation, chemical bath deposition, spin coating etc, have been employed for the preparation of CTS [38].

However, mathematical calculations of a CdTe–FeSi_2_ tandem solar PV cell have been conducted in this study. The device parameters are calculated by considering material properties such as energy bandgap, diffusion length, and doping concentration. The variation in electrical characteristics of the presented tandem solar cell with varying the concentration of dopants, thickness, and the energy bandgap have been extensively studied.

## 2 Methodology for designing and simulating the device

**Fig. 1:**
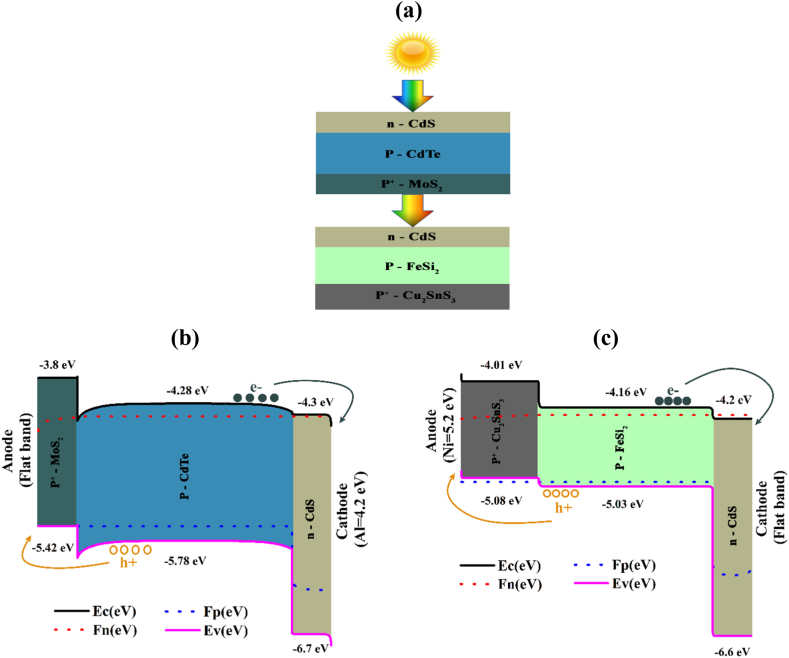
(a) Configuration of the CdTe–FeSi_2_ 2-terminal PV device, (b) Electronic structure of the upper cell, and (c) Electronic structure of the lower cell.

Fig. 1(a) illustrates the graphic representation of the raised CdTe–FeSi_2_ based tandem cell, where the energy band configurations of the upper and lower cells describe by Fig. 1(b) and (c), respectively. The proposed PV solar cell structure which consists of CdS as the window layer for both the upper and lower cells, possessing a bandgap of 2.4 eV and an electron affinity of 4.3 eV MoS_2_ has been chosen as the top cell BSF layer with a bandgap and electron affinity of 1.62 eV and 3.8 eV, and Cu_2_SnS_3_ (CTS) incorporated as the (BSF) layer in the lower cell with 1.07 eV energy gap and electron affinity of 4.01 eV.

CdTe has featured a 1.5 eV bandgap and a 4.28 eV electron affinity, while the bandgap and electron affinity of FeSi_2_ are 0.87 eV and 4.16 eV, respectively. Because of having higher bandgap, CdTe has been considered as the layer of absorption of the upper cell, while owing to its lower bandgap, FeSi_2_ has been comprised as the bottom cell absorber layer. Here, both CdTe and FeSi_2_ can potentially build a p-n junction with CdS. In opposition, MoS_2_ forms a p-p^+^ hetero-junction with CdTe, while Cu_2_SnS_3_ (CTS) forms a p-p^+^ hetero-junction with FeSi_2_ in the up and down cell, in turn. An optimal, slender tunnel junction connecting the upper and lower cells with monolithic architecture has been assumed for the electrical linkage. In this scenario, the impact of parasitical light absorption and electrical diminutions has been assumed to be insignificant or perfect. The CdTe top cell independently provides a PCE of 26.13%, while the independent FeSi_2_ bottom cell produces a PCE of 35.25%. These material's lattice parameters may inaugurate imperfections at the interfaces of CdS/CdTe and CdS/FeSi_2_ due to threading dislocation density (TDD). Moreover, the model also considers defects generated during fabrication, including bulk defects.

The proposed CdTe–FeSi_2_ based double-junction two-terminal tandem cell's simulation is conducted running SCAPS 1D simulator (version 3.3.07) at a working temperature of 300 K [[39], [40], [41]]. The simulation software was developed by Professor M. Burgelman and his research team at the University of Gent in Belgium. It primarily addresses the resolution of Poisson's equations for hole and electron continuity.

(1) $${(\text{Poisson}}^{\prime }s\;\text{equation})\;\frac{\partial^{2}\Psi }{\partial x^{2}}+\frac{q}{\varepsilon }\left[p\left(x\right)-n\left(x\right)+N_{D}-N_{A}+\rho_{p}-\rho_{n}\right]=0$$

(2) $$\left(\text{Hole}\;\text{continuity}\;\text{equation}\right)\;\frac{1}{q}\frac{\partial J_{p}}{\partial x}=G_{op}-R\left(x\right)$$

(3) $$\left(\text{Electron}\;\text{continuity}\;\text{equation}\right)\;\frac{1}{\;q}\frac{\partial J_{n}}{\partial x}=-G_{op}+R\left(x\right)$$

In this context, ε denotes the permittivity, q for the charge of electron, N_A_ and N_D_ stand for the concentrations of charged acceptors and donors. J_p_ and J_n_ are used to express the current densities of holes and electrons, respectively. Additionally, Ψ is the symbol for electric potential, G_op_ corresponds to the overall generation rate, R symbolizes the total recombination rate, p represents the concentration of free holes, and n denotes the density of free electrons. ρ_p_ and ρ_n_ refer to the spatial distribution or arrangement of holes and electrons, in turn.

The succeeding equations of drift-diffusion can calculate the conductivity behavior of holes and electrons within the semiconductor layers:

(4) $$J_{p}=-\frac{\mu_{p}p}{q}\frac{{\partial E}_{Fp}}{\partial x}$$

(5) $$J_{n}=-\frac{\mu_{n}n}{q}\frac{{\partial E}_{Fn}}{\partial x}$$

Here, E_Fp_ and E_Fn_ denote the quasi-Fermi levels for carriers in p-type and n-type materials, respectively. Additionally, ***μ***_p_ represents the mobility of holes, and ***μ***_n_ indicates the mobility of electrons.

**Mechanism of top cell:** To simulate the top cell, solar irradiation at 1 sun intensity level with a power density of 1000 W/m^2^ and the standard air mass (AM) of 1.5G solar spectrum has been used. The coefficient of absorption is calculated by obeying the next equation [42],

(6) $$\alpha \left(\lambda \right)=\alpha_{0}\left(\sqrt{\frac{\left(hc/\lambda \right)-E_{g}}{KT}}\right)$$

In the above equation, $\alpha_{0}$ is representing the material absorption coefficient, K_B_ stands for the Boltzmann constant, c signifies the speed of light and T stands for absolute temperature.

The top cell's electrical characteristics are defined by parameters such as the carrier diffusion length (L_d_), luminescence efficiency (Φ), and (FF). The short-circuit current is influenced by the absorption of incoming light and the probability of carrier collection ($f_{c}$) [42],

(7) $$J_{SC}=f_{c}\int_{0}^{\lambda_{m}}q\varphi_{\text{AM}1.5G}\left(\lambda \right)A^{1}\left(\lambda \right)d\lambda$$

where, φ_AM1.5G_(λ) denotes the flux of incident photons and A^1^ is the single pass absorption denoted by,

(8) $$A^{1}=1‐e^{-\left(\alpha w\right)}$$

At the same time, carrier collection probability,

(9) $$f_{c}=\frac{e^{\left(\left(\lambda 0_{2}\right)/2\right)}-1}{\left(\left(\lambda 0_{2}\right)/2\right)}$$

here,

(10) $$\lambda_{0_{2}}=\frac{qV_{bi}}{2kT}\;-\sqrt{{\left(\frac{W}{L_{d}}\right)}^{2}+{\left(\frac{{qV}_{bi}}{2kT}\right)}^{2}}$$

In the previous equation V_bi_ is the built in potential [42].

**Mechanism of bottom cell:** The current in short circuit for the bottom cell can be calculated by Ref. [42],

(11) $$J_{SC}=0.978\int_{0}^{\lambda_{m}}q\varphi_{\text{AM}1.5G}\left(\lambda \right)S\left(\lambda \right)A^{2}\left(\lambda \right)d\lambda$$

Wherein S(λ) is the spectrum after filtration used for the bottom cell. The equation used for spectrum filtering is as follows:

(12) $${S\left(\lambda \right)=S}_{0}\;\exp\;\left(\sum_{i}^{n}-\left(a\left(\lambda \right)\times d_{{mat}_{i}}\right)\right)$$

In this context, we represent the incoming AM 1.5G and the modified spectrum as S_0_(λ) and S(λ), respectively. Each specific compound is denoted as “mat_i_,” where i corresponds to 1 for CdS, 2 for CdTe, and 3 for MoS_2_.

Equation (11) lacks consideration for the wavelength-specific variations in the internal quantum efficiency. Nevertheless, it remains sufficiently precise for the context of this research.

The parameters for the breadth, coefficient of absorption (α(λ)), and the cells number are denoted as d, α(λ), and n, in turn. Equations (13), (14) below describe the quantum efficiency (QE) for both the front and back cells [43].

(13) $${QE}_{top}\left(\lambda \right)=A_{CdTe}\left(\lambda \right)$$

(14) $${QE}_{bottom}\left(\lambda \right)={QE}_{Fe{Si}_{2\;}\;}\left(\lambda \right)\times T\left(\lambda \right)$$

In this context, A and T are used to denote absorption and transmission, accordingly. The T is determined based on the relationship between S(λ) and S_0_(λ). The QE for the lower cell, referred to separately, is represented as ${QE}_{Fe{Si}_{2\;}}$.

From eq (11) one can derive the value of open circuit voltage as follows,

(15) $$V_{OC}=\frac{KT}{q}\ln\left(\left(\frac{J_{sc}}{J_{o}}\right)+1\right)$$

From the V_oc_, the FF can be estimated by Ref. [44],

(16) $$\text{FF}=\frac{v_{oc-\ln\left(v_{oc}-0.72\right)}}{v_{oc}}$$

with $v_{oc}=\frac{V_{oc}}{nK_{B}T/q}$, herein, n the diode ideality factor.

Finally by utilizing all of the equations, the final outcome, the efficiency can be calculated by,

(17) $$\eta =\frac{J_{sc}V_{oc}FF}{P_{in}}$$

Utilizing a script file SCAPS simulate tandem solar cells. Once executed, the script instructs SCAPS to perform simulations based on input parameters. Notably, it can incorporate iterative optimization routines to fine-tune device parameters for enhanced efficiency. Particularly useful for tandem cells, the script enables seamless design, simulation, and analysis within SCAPS 1D [45].

The script file which have used in this study is provided in the supplementary file.

The relevant physical characteristics for CdS, CdTe, FeSi_2_, MoS_2_, and Cu_2_SnS_3_(CTS) have been extracted from existing literature [18,20,46,47]. For optical absorption data, we've utilized the SCAPS optical E_g_-sqrt model with its default settings. Table 1 presents the physical attributes and simulation parameters for the envisioned solar device based on CdTe and FeSi_2_, while Table 2 contains information about the interface parameters.

**Table 1:** Input variables utilized in CdTe–FeSi_2_ Tandem solar cells.

Parameters	*n-*CdS [46]	*p*-CdTe [18]	*p*-FeSi_2_ [20]	*p* + -MoS_2_ [46]	*p* + -Cu_2_SnS_3_ [47]
Types of layer	Window	Absorber	Absorber	BSF	BSF
Thickness (μm)	0.1	0.55	0.45	0.1	0.2
Energy bandgap, E_g_ [eV]	2.4	1.5	0.87	1.62	1.07
Affinity of electrons, χ [eV]	4.3	4.28	4.16	3.80	4.01
Relative dielectric constant	10	10.500	22.50	10.070	13.60
CB effective DOS [cm^−3^]	2.2 × 10^18^	2.9 × 10^17^	5.6 × 10^19^	2.8 × 10^19^	2.2 × 10^18^
VB effective DOS [cm^−3^]	1.8 × 10^19^	5.2 × 10^18^	2.08 × 10^19^	1.0 × 10^19^	1.8 × 10^19^
Velocity of thermal electrons (cms^−1^)	1.0 × 10^7^	1.0 × 10^7^	1 × 10^7^	1.0 × 10^7^	1.0 × 10^7^
Velocity of thermal holes (cms^−1^)	1.0 × 10^7^	1.0 × 10^7^	1.0 × 10^7^	1.0 × 10^7^	1.0 × 10^5^
Mobility of electron, μ_n_ [cm^2^V^−1^s ^−1^ ]	1.0 × 10^2^	3.2 × 10^2^	1.0 × 10^2^	1.2 × 10^1^	1.0 × 10^2^
Mobility of hole, μ_p_ [cm^2^V^−1^s^−1^ ]	2.5 × 10^1^	4 × 10^1^	2.0 × 10^1^	2.80	2.5 × 10^1^
Donor concentration, N_D_ [cm^−3^ ]	1.0 × 10^18^	0	0	0	0
Acceptor concentration, N_A_ [cm^−3^ ]	0	1.0 × 10^16^	1.0 × 10^18^	1.0 × 10^19^	1.0 × 10^20^
Type of defect	Single Acceptor	Neutral	Single Acceptor	Neutral	Single Donor
Energetic distribution	Gaussian	Single	Gaussian	Single	Gaussian
Energy level w. r. to Reference (eV)	0.7	0.6	0.6	0.6	0.65
Total defects, N_t_ [cm^−3^]	1.0 × 10^14^	1.0 × 10^14^	1.0 × 10^13^	1.0 × 10^14^	1.0 × 10^14^

**Table 2:** Interface input parameters used in this simulation.

Parameter	Topmost cell		Bottommost cell	
	*p*-CdTe/*n*-CdS	*p*-CdTe/*p* + -MoS_2_	*p*- FeSi_2_/*n*-CdS	*p*-FeSi_2_/*p* + - Cu_2_SnS_3_
Defect type	neutral	neutral	neutral	neutral
Total density (1/cm^2^)	1.0 × 10^11^	1.0 × 10^11^	1.0 × 10^10^	1.0 × 10^10^

The parameters for the top and bottom cells, including defect types and total density in Table 2, have been selected based on literatures [20,46,47] and SCAPS default settings. These parameters have been carefully determined to ensure consistency and reliability in the simulation approach.

## 3 Results and discussion

Different attributes such as thickness, doping, and the density of defects of top and bottom cells are varied to observe the influence on the performance of the solar cell in tandem configuration. By varying these parameters, an optimized tandem configuration with optimized parameters is obtained for maximum efficiency. The device's design results in greater voltage at open circuit (V_OC_), the current density at short circuit (J_SC_), and the total efficiency when contrasted with traditional homojunction photovoltaic cells utilizing crystalline silicon [[48], [49], [50], [51]].

### 3.1 Role of the thickness of the upper and lower absorbers in PV parameters

**Fig. 2:**
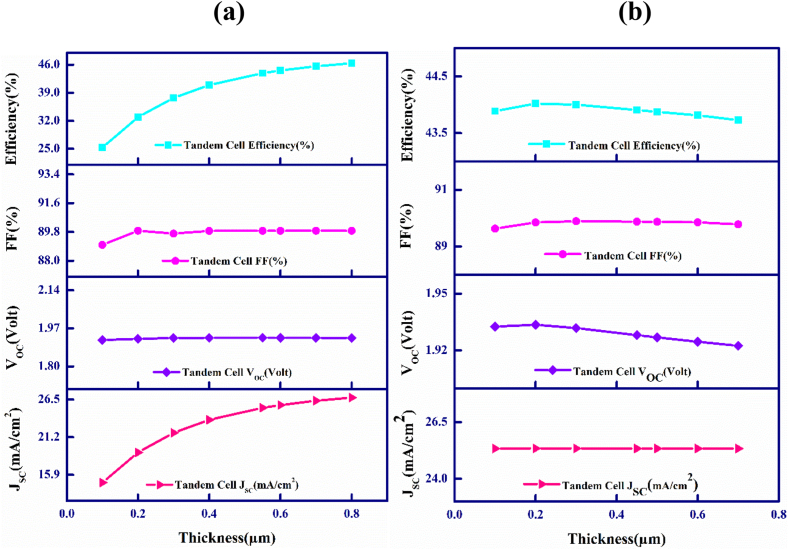
Width vs. PV characteristics of the upper and lower cells for (a) CdTe and (b) FeSi_2_ layers of the CdTe–FeSi_2_ tandem cell.

Fig. 2(a) and (b) describe the outcome of variation in thicknesses of the *p*-CdTe and *p*-FeSi_2_ base materials of the proposed tandem structure. Fig. 2(a) depictures that with the variation in thickness of CdTe the layer for absorption between 0.1 and 0.8 μm, an avoidable variation of V_OC_ and is fixed at around 1.92 V. Nevertheless, increasing the thickness further might lead to a rise in recombination current, thus reducing the V_OC_ [52]. Contrarily, J_SC_ increases continuously between 14.82 mA/cm^2^ and 26.78 mA/cm^2^ in the observed width range. The amelioration of J_SC_ is noteworthy as higher amounts of photons are collected with the lengthening of the layer responsible for absorbing [53]. The fill factor initially increases to 89.88%, then decreases to 89.7% before increasing again and remaining constant at 89.87%. Nonetheless, the PCE demonstrates an advancement from 25.29 to 46.39% as it obeys the positive change of J_SC_. An optimal breadth of 0.55 μm is chosen for the CdTe layer for further analysis. Leading to PCE of 43.91% having V_OC_ ∼1.928 V and J_SC_ ∼25.34 mA/cm^2^ and fill factor of 89.88%, accordingly.

Fig. 2(b) emphasizes how the photovoltaic performance varies with the width of the FeSi_2_ layer ranging between 0.1 and 0.8 μm. The figure illustrates that with increasing thickness, there is a negligible decrease in V_OC_, which stabilizes at around 1.928 V. On the contrary, a constant J_SC_ of 25.34 mA/cm^2^ is observed. The fill factor varies slightly between 89.63 and 89.89%. Furthermore, The PCE initially increases to 44.02% but then decreases to 43.73% due to a drop in the voltage when there is an open circuit.

We can infer that with the enhancement of width of the lower absorber layer, the PV cell's output decreases slightly. For further studies, it has been ensured that the bottom layer has a consistent width of 0.45 μm, which is considered optimal for its intended purposes. This measurement is determined through aligning the current within the upper and lower sub-cells of the CdTe–FeSi_2_ dual-junction two-edged multijunction PV device as displayed in Table 1.

### 3.2 Influence of carrier density in the upper and lower absorbers on PV parameters

**Fig. 3:**
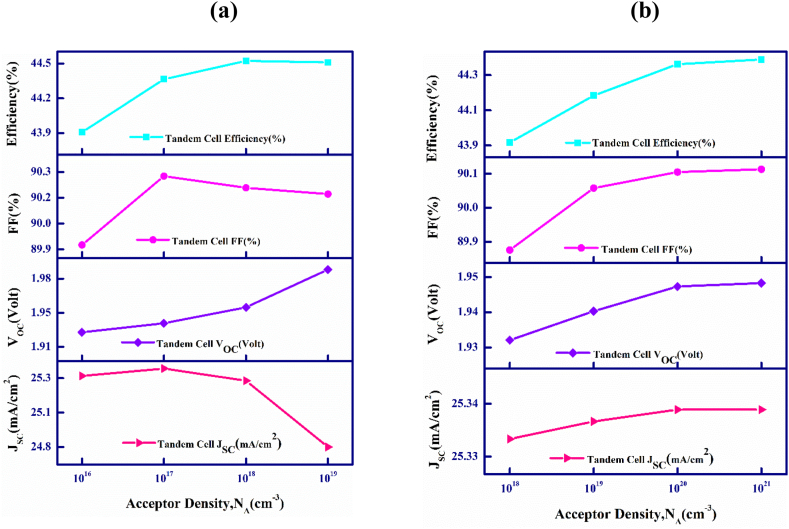
(a) Output parameters plotted against top absorber acceptor density, and (b) Output parameters plotted against bottom absorber acceptor density in CdTe–FeSi_2_ tandem cell.

The acceptor values, N_A_ in CdTe and FeSi_2_ absorbers have been varied from 1 × 10^16^ to 1 × 10^19^ cm^−3^ for the upper and 1 × 10^18^ to 1 × 10^21^ cm^−3^ for the bottom cells of the proposed CdTe–FeSi_2_ multiple-stacked PV cell that is displayed in Fig. 3(a) and (b).

Fig. 3(a) exhibits the difference in the tandem cell activities when the carrier density fluctuates in the top CdTe material. As observed, the J_SC_ initially increases to 25.39 mA/cm^2^ and then decreases slightly to 24.84 mA/cm^2^ with the amelioration of the impurity levels. This is so as to the higher losses results from recombination at heavy doping density, instigates sudden drop of J_SC_ [54,55]. Furthermore, the V_OC_ value increases from 1.92 to 1.98 V, indicating an upward trend. Similarly, the fill factor initially follows this positive tradition and increases from 89.88% to 90.28% up to 10^17^ doping density but later continues to decrease to 90.17% with a further increase in doping density. Nevertheless, the device's efficiency improves from 43.91 to 44.56% with the rise in doping density up to 10^18^, then decreases slightly to 44.55% as the doping is increased further to 10^19^, following the decrease in J_SC_.

Fig. 3(b) delineates the photovoltaic output parameters of the tandem solar cell owing to the variation in carrier density of the FeSi_2_ lower absorbing material. It is observed that all the PV parameters show an increase in value with the rise in doping density. However, J_SC_ and V_OC_ increase inappreciably from 25.34 to 25.342 mA/cm^2^ and 1.928–1.944 V, respectively. The fill factor and PCE increase quite a margin from 89.88 to 90.11% and from 43.91 to 44.4%, specifying that the proposed tandem device performance will enhance if the doping is increased within the FeSi_2_ bottommost absorbing layer.

### 3.3 Influence of defects in both the upper and lower absorbers on cell outputs

**Fig. 4:**
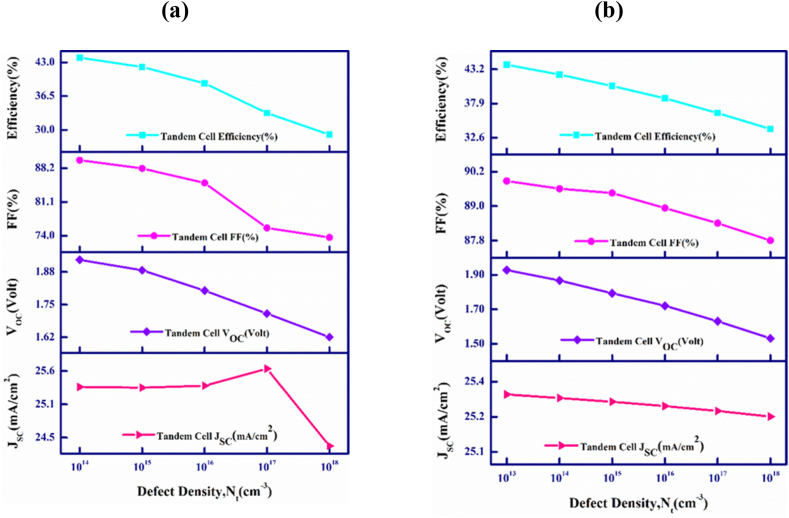
(a) Output parameters plotted against defects amount of upper absorber, and (b) Output parameters plotted against defects of lower absorber in CdTe–FeSi_2_ solar cell.

Fig. 4(a) and (b) visualize the outcome of variation in the density of defect of the proposed tandem device's *p-*CdTe and *p-*FeSi_2_ absorber layer. The defect density, N_t_ in CdTe and FeSi_2_ absorber layers have been varied in the range from 1 × 10^14^ to 1 × 10^18^ cm^−3^ and 1 × 10^13^ to 1 × 10^18^ cm^−3^, respectively. The effectiveness of the solar PV cell's output is significantly influenced by the imperfections found in its various cell layers [56].

In Fig. 4(a), the PV output performance of the CdTe–FeSi_2_ photovoltaic cell is shown to be dependent with the variation in defects within the CdTe top material. As the level of defect in the top CdTe base layer increases, all performance parameters decline, except for J_SC_, which also decreases after an initial rise. The values of V_OC_, FF, and PCE shift from 1.92 to 1.62 V, 89.88 to 73.63%, and 43.91 to 29.08%, respectively. The reduction in the V_OC_ value directly results from an increase in reverse saturation current due to higher defects [57,58]. However, the value of J_SC_ has obeyed a levelled value of 25.34 mA/cm^2^ up to 10^16^ cm^−3^. After crossing this range, the amount of J_SC_ initially depictures an increment to 25.64 mA/cm^2^ with an increment of ∼0.3 mA/cm^2^ at a defect of 10^17^ cm^−3^ and then decreases to 24.36 mA/cm^2^ with more increment of the concentration of defect. This effect can be explained by the equivalent circuit model of the solar cell represented by a diode. If defects are found within the depletion layer, an advancement in defects can lead to the formation of conduction pathways and affect shunt resistances. In that case, the defects behave like dopants, altering the profile of doping and lessening the depletion region of the heterojunction. Therefore, J_SC_ does not change significantly with defects up to the level 10^17^ cm^−3^ [59]. However, if defects appear out of the depletion layer, the elevation of the defects will rise the non-radiative recombination rates which function as the traps for the photo-generated current. Therefore, short circuit current of the device decreases at higher defects [60].

Fig. 4(b) shows the impact of the variation of the defects in the FeSi_2_ bottom layer on the PV output activities of the CdTe–FeSi_2_ based solar cell. The rise in defect density within the lower FeSi_2_ absorber layer leads to a consistent decline in all performance parameters. As defect density increases from 10^13^ to 10^18^ cm^−3^, the J_SC_ experiences a slight drop from 25.34 mA/cm^2^ to 25.23 mA/cm^2^. Simultaneously, the V_OC_ exhibits a noticeable decrease, moving from 1.928 to 1.532 V. As the concentration of defect is increased, it elevates the rate of the recombination of photo-carriers within the FeSi_2_ absorber layer concurrently decreases the carrier lifetime, resulting in the degradation of the tandem device's output performance. Additionally, the increase in recombination centers within the FeSi_2_ layer contributes to a demotion in parallel resistance, thereby diminishing the V_OC_ of the cell [61]. Likewise, the FF and PCE decline from 89.88 to 87.80% and 43.91 to 33.94%, respectively.

This emphasizes that the proposed tandem device's performance will deteriorate if the defect density is increased in both the CdTe top and the FeSi_2_ bottom absorber layers.

### 3.4 Impact of window and BSF layers

#### 3.4.1 The role of window layer

**Fig. 5:**
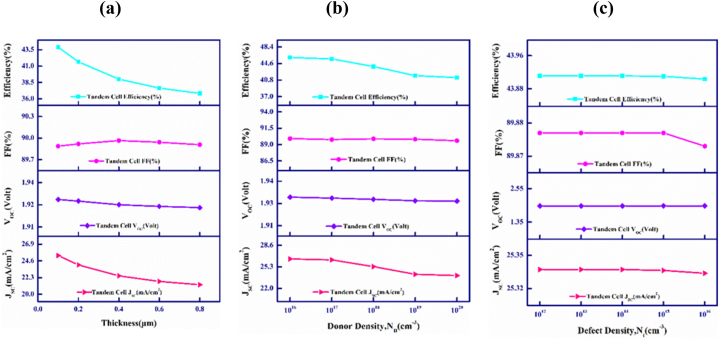
Variations in CdTe–FeSi_2_ device's outputs with upper cell's window's (a) Breadth, (b) Donor density, N_D_, and (c) Defects, N_t_.

Fig. 5(a), along with 5(b) and 5(c) illustrate the outcomes of adjusting the thickness, donor density (N_D_) and defect density (N_t_) of the upper window layer on the PV elements of the CdTe–FeSi_2_ PV solar cell.

Fig. 5(a) delimitates that the variation of the width of the top cell CdS window layer influences the performance and output parameters of the proposed tandem cell, ranging from 0.1 to 0.8 μm. The current in short circuit as well as PCE are observed to reduce with the increase in the width from 25.34 to 21.32 mA/cm^2^ and 43.91 to 36.83%. This occurs due to the increase in unwanted absorption, which restricts shorter-wavelength photons from reaching the absorber layer [18]. However, the FF and V_OC_ depict little affection with the alteration of the width of the window layer that practically remains constant at 89.9% and 1.92 V, respectively. Because of the combination of elevated carrier mobility and a broad bandgap, variations in the thickness of the front layer have limited impact on the photovoltaic components [62].

Fig. 5(b) visualizes the outcome of variation in donor density (N_D_) of the upper cell CdS window layer of the proposed multi-junction cell. The donor concentration of the window layer has been manipulated between 1 × 10^16^ and 1 × 10^20^ cm^−3^. It is observed that the values of FF and V_OC_ stay unchanged as the doping density rises. On the contrary, the current in short circuit as well as PCE are observed to abate with the increase in doping density between 26.51 and 23.97 mA/cm^2^ and 46 to 41.4%. It is evident that changes in doping concentration strongly impact J_SC_ and PCE, resulting in a decrease in their values. However, V_OC_ and FF show less sensitivity to changes in impurity level. At elevated doping concentrations, free carrier recombination is heightened results the decline in J_SC_ and PCE [57].

Fig. 5(c) illustrates how the photovoltaic performance changes with the manipulation of the concentration of defect (N_t_) of the supernal cell CdS window layer, ranging from 1 × 10^12^ to 1 × 10^16^ cm^−3^. It is clear by observing the diagram that all performance parameters remain nearly consistent with the discrepancy of the density of defect of the uppermost window layer. Although, it is observed that defects in the upper cell's window layer hardly affect the photovoltaic parameters of the proposed device. Nevertheless, additional increase in imperfection density lead to an elevation in dark current, potentially having a significant impact on device performance [63].

**Fig. 6:**
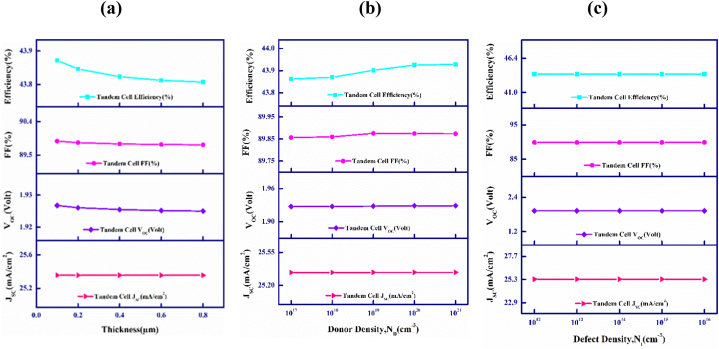
Variations in CdTe–FeSi_2_ device's outputs with lower cell's window's (a) Breadth, (b) Donor density, N_D_, and (c) Defect density, N_t_.

Fig. 6(a), (b), and (c) describe the outcome of altering the bottom cell window layer's width, donor density (N_D_) and defect density (N_t_) on the PV attributors of the CdTe–FeSi_2_ PV photovoltaic cell.

Fig. 6(a) exhibits how the deviation in width of the lowermost cell CdS window layer influences the performance and output parameters of the multi-junction cell. The width has been varied in the span of 0.1–0.8 μm. As observed, all performance parameters practically stay unchanged with the discrepancy in the breadth of the bottom front layer. The J_SC_, V_OC_, and FF remain consistent at 25.34 mA/cm^2^, 1.92 V, and 89.8%, respectively with PCE slightly decreasing from 43.91 to 43.81%.

Fig. 6(b) visualizes the outcome of alteration in donors (N_D_) of the lowermost CdS layer of the proposed tandem device. The donor density has been altered from 1 × 10^17^ to 1 × 10^21^ cm^−3^. The J_SC_ and V_OC_ remain unchanged at 25.34 mA/cm^2^ and 1.92 V. Contrarily, the FF as well as PCE enhance meagerly from 89.86 to 89.87% and 43.88–43.92%, in turn.

Fig. 6(c) describes how the photovoltaic performance alters with the alteration of the density of defect (N_t_) of the bottom cell CdS window layer, spanning from 1 × 10^12^ to 1 × 10^16^ cm^−3^. The figure shows that all the outputs are leveled off with the discrepancy of the concentration of defect of the lower cell window layer.

Due to the higher mobility of carriers and the length of diffusion of the CdS semiconductor, changes in the breadth, doping, and defects of the CdS layer do not substantially affect device performance [63].

#### 3.4.2 MoS_2_ and Cu_2_SnS_3_ (CTS) BSF layers impact on tandem cell

In this segment, we explore the alterations in the performance and output characteristics of the CdTe–FeSi_2_ tandem solar cell when modifying the BSF layer's width, carrier density (N_A_), and defect density (N_t_) in the upper and lower cells.

**Fig. 7:**
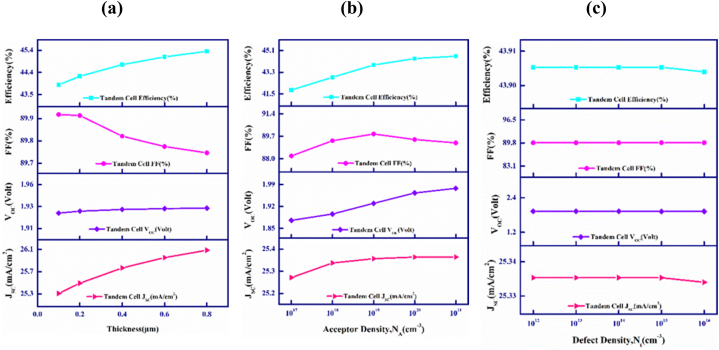
Variations in CdTe–FeSi_2_ device's outputs with the upper's BSF's (a) Breadth, (b) Donor density, N_D_, and (c) Defect density, N_t_.

Fig. 7 delineates the result of changing the thickness, carrier and defect density, of the MoS_2_ BSF layer in the top cell on the outputs of the CdTe–FeSi_2_ based PV cell.

Fig. 7(a) describes how the variation in the width of the upper cell MoS_2_ BSF layer influences the performance and output parameters of the proposed tandem cell, ranging from 0.1 to 0.8 μm. It is noted that the J_SC_, V_OC_, and efficiency all show an upward trend from 25.34 to 26.12 mA/cm^2^, 1.928–1.933 V, and 43.91–45.32%, in sequence as the thickness increases However, FF falls from 89.88 to 89.76%. However, the decision to limit the thickness range at 0.1 μm has been made to balance photon absorption and transmission within the tandem solar cell structure. Practical factors such as material quality, fabrication ease, and cost have also influenced this decision. The analysis has revealed that increasing thickness initially improved efficiency by enhancing light trapping and carrier collection. However, efficiency has significantly dropped at high thickness levels compared to low thickness levels. That's why we have observed thickness only up to 0.8 μm [64].

Fig. 7(b) depicts the outcome of variation in the concentration of acceptor (N_A_) of the upper cell MoS_2_ BSF layer of the proposed multi-junction device. The acceptor density has been altered in the boundary from 1 × 10^17^ to 1 × 10^21^ cm^−3^. The J_SC_, V_OC,_ and PCE are observed to enhance with the increase in doping density from 25.28 to 25.34 mA/cm^2^, 1.87–1.97 V, and 41.82–44.64%. On the contrary, the FF ameliorates from 88.22 to 89.88 before decreasing to 89.21%. The highly doped BSF layer generates a potential barrier that pushes minority carriers back into the bulk of the cell. This promotes better collection of photo-generated carriers, resulting in improvements in PV parameters [65].

Fig. 7(c) exhibits how the photovoltaic performance changes when the density of defect in the upper cell's BSF layer fluctuates ranging between 1 × 10^12^ and 1 × 10^16^ cm^−3^. The figure implies that all performance parameters remain constant though the density of defect in the top cell BSF layer changes, which elaborates that the increasing density of defect practically has no influence on the device's activities. However, an excess amount of defects may advance the reverse current, which poses a threat to the functionality of the cell [63].

The tandem cell's output performance may vary slightly depending on the alteration in the top cell MoS_2_ layer. This can be attributed to the reduction in the recombination rate at the surface caused by doping. In consequences, the junction's built-in voltage is enhanced, which affects the current [66,67].

**Fig. 8:**
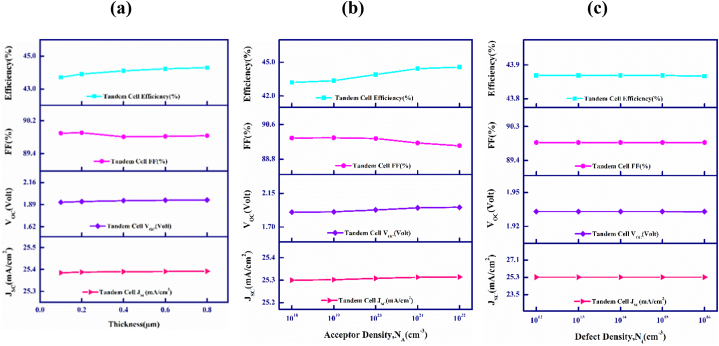
Variations in CdTe–FeSi_2_ cell's outputs with lower cell's BSF's (a) Breadth, (b) Donor density, N_D_, and (c) Defect density, N_t_.

Fig. 8(a), (b), and (c) represent the impact of the variation in the bottom cell Cu_2_SnS_3_ (CTS) BSF layer's thickness, acceptor density (N_A_) and defect density (N_t_) on the PV parameters of the CdTe–FeSi_2_ based PV solar cell. It is observed that with the variation of those parameters mentioned above in the bottom cell CTS BSF layer, the general effectiveness of the PV solar cell does not change and remains constant.

### 3.5 Maximized device outcomes

**Fig. 9:**
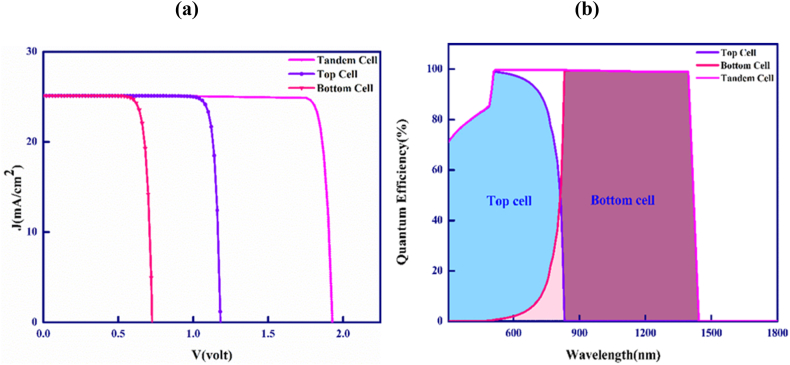
(a) Current (J)-voltage (V) and (b) quantum efficiency plots of CdTe–FeSi_2_ 2-terminal dual-junction tandem cell.

Fig. 9(a) exhibits the current-voltage (J-V) plots for the upper and lower cells of the CdTe–FeSi_2_ based tandem cell. To achieve optimal performance in the tandem cell, it is necessary for both the upper and lower cells to have an equal J_SC_ since they are directly interconnected. The point of current matching is determined by adjusting the breadths of both the absorber layers in the CdTe top cell and the FeSi_2_ bottom cell. Furthermore, after thinning the top absorber layer, a simulated analysis is performed on the spectrum of passing light through the upper sub-cell and a filtered spectrum has obtained. This filtered spectrum has later employed as the incoming light spectrum to illuminate the lower sub-cell for further simulations. At the current matching point, the optimum widths for both the upper CdTe and lower FeSi_2_ absorber layer are 0.55 and 0.45 μm while the matching J_SC_ is 25.13 mA/cm^2^. The optimized thickness for the CdS window layer is 0.1 μm. In contrast, the optimized thicknesses for MoS_2_ and CTS BSF layer are 0.1 and 0.2 μm. The optimal concentration of defects of the window, upper and lower absorbers, and the BSF layers are 10^14^, 10^14^, 10^13^ and 10^14^ cm^−3^. Similarly, the optimized doping concentrations are 10^18^, 10^16^, and 10^18^ cm^−3^ for the window, upper, and lower absorber layers. However, the optimized concentrations of impurity for MoS_2_ and CTS BSF layers are 10^19^ and 10^20^ cm^−3^.

The variation in the quantum efficiency (QE) of the CdTe–FeSi_2_ based tandem solar cell concerning the wavelength of the incident spectrum of sunlight is depictured in Fig. 9(b). The quantum efficiency (QE) of a photovoltaic cell is expressed as the proportion of the total collected charges to the number of incident photons. A quantum efficiency (QE) of 100% is attainable when every incident photon is converted into electric charges. The QE is assessed in relation to the wavelength of light [54,60]. However, quantum efficiency (QE) reaches 100% only at a specific wavelength of light [60]. Noticeably, the QE is 87% at 740 nm and falls to 0% around 832 nm for the CdTe upper layer for absorption. Contrarily, illuminating the FeSi_2_ lower absorber layer with the filtered spectrum results in a QE of 12.6% at 740 nm and almost a QE of 99% from 832 to 1395 nm. Then, the QE falls quickly to zero around 1428 nm. This emphasizes that the CdTe–FeSi_2_ tandem solar PV cell demonstrates an impressive quantum efficiency (QE) within both the visible and infrared spectrum up to the wavelength of 1395 nm.

## 4 Conclusion

The study has focused on the operational effectiveness of an enormously efficient double-junction solar cell based on CdTe and FeSi_2_, incorporating CdS as the window layer and MoS_2_ and CTS as back surface field (BSF) layers. The SCAPS-1D simulator is used to investigate and optimize various parameters, including thickness, impurity concentration and defect density to maximize the tandem cell's efficiency. Furthermore, a detailed analysis explores the effects of width, carrier, and defect density across various layers, like CdTe and FeSi_2_ absorber layers as well as CdS window and MoS_2_, CTS BSF layers. The simulation results demonstrate the potential to achieve a high V_OC_ of 1.928 V and an efficiency of 43.91%. These findings also indicate the J_SC_ of 25.338 mA/cm^2^ and the FF of 88.88%. These results suggest the practical feasibility of fabricating high-performance CdTe–FeSi_2_ double-junction tandem solar cells for efficient solar energy conversion.

## Data availability statement

Data will be made available on request.

## CRediT authorship contribution statement

**Mehedi Hasan Tonmoy:** Writing – original draft, Investigation, Formal analysis, Data curation. **Sheikh Noman Shiddique:** Writing – original draft, Investigation, Formal analysis, Data curation. **Ahnaf Tahmid Abir:** Writing – original draft, Validation, Formal analysis, Data curation. **Jaker Hossain:** Writing – review & editing, Writing – original draft, Validation, Supervision, Methodology, Investigation, Formal analysis, Data curation, Conceptualization.

## Declaration of competing interest

The authors declare that they have no known competing financial interests or personal relationships that could have appeared to influence the work reported in this paper.
