# Supplementary material for: Design and optimization of a high efficiency CdTe–FeSi2 based double-junction two-terminal tandem solar cell
Source: Heliyon. 2024 Mar 12;10(6):e27994. doi: 10.1016/j.heliyon.2024.e27994 (PMC10958414; doi:10.1016/j.heliyon.2024.e27994)
Supplement: Multimedia component 1 [file mmc1.docx]

//Script for SCAPS Tandem solar cell

clear all

load definitionfile CdS-CdTe-MoS_2_ Top Cell.def

calculate singleshot

get iv xy //for top cell:v is stored in xvector,i in yvector

get characteristics.jsc yvalue

set scriptvariable.yvalue yvector[0]

math scalarabs yy

load definitionfile CdS-FeSi_2_-CTS Bottom Cell.def

calculate singleshot

get iv zu //for bottom cell:v is stored in uvector,i in vvector

get characteristics.jsc uvalue

set scriptvariable.vvalue 0.002

math scalarsubtract vvy

set scriptvariable.wvalue 0

math filllinear vvector vvalue wvalue 100

set scriptvariable.maxiteration nv

set scriptvariable.nw nv

math fillconstant w 0 nw

//start the loop:vary Jtandem

loop start

//find the voltage of the top cell at this Jtandem (by interpolation); set it in xvalue

set scriptvariable.yvalue vvector[loopcounter]

math interpolate xXyY

//find the voltage of the bottom cell at this Jtandem (by interpolation); set it in zuvalue

set scriptvariable.uvalue vvector[loopcounter]

math interpolate zZuU

//do the series connection:add the voltage of top and bottom cell the result is in xvalue

math scalaradd xxz

set scriptvariable.wvector[loopcounter] xvalue

loop stop

set scriptvariable.xname Vtop(V)

set scriptvariable.yname Jtop(mA/cm2)

set scriptvariable.zname Vbottom(V)

set scriptvariable.uname Jbottom(mA/cm2)

set scriptvariable.wname Vtandem(V)

set scriptvariable.vname Jtandem(mA/cm2)

show scriptvariables

plot draw wv

plot draw xy

plot draw zu

//ectracting the efficiency parameters of the tandem,and place themin the scalars xvalue

math characteristics.voc xwv

math characteristics.jsc ywv

math characteristics.ff zwv

math characteristics.eta uwv

math characteristics.vmpp vwv

math characteristics.jmpp wwv

show scriptvariables
